# Chimeric Antigen Receptor T Cell Therapy in Hematology

**DOI:** 10.4274/tjh.2015.0049

**Published:** 2015-12-03

**Authors:** Pınar Ataca, Önder Arslan

**Affiliations:** 1 Ankara University Faculty of Medicine, Department of Hematology, Ankara, Turkey

**Keywords:** Chimeric antigen receptor T cell, Hematological malignancies

## Abstract

It is well demonstrated that the immune system can control and eliminate cancer cells. Immune-mediated elimination of tumor cells has been discovered and is the basis of both cancer vaccines and cellular therapies including hematopoietic stem cell transplantation. Adoptive T cell transfer has been improved to be more specific and potent and to cause less off-target toxicity. Currently, there are two forms of engineered T cells being tested in clinical trials: T cell receptor (TCR) and chimeric antigen receptor (CAR) modified T cells. On 1 July 2014, the United States Food and Drug Administration granted ‘breakthrough therapy’ designation to anti-CD19 CAR T cell therapy. Many studies were conducted to evaluate the benefits of this exciting and potent new treatment modality. This review summarizes the history of adoptive immunotherapy, adoptive immunotherapy using CARs, the CAR manufacturing process, preclinical and clinical studies, and the effectiveness and drawbacks of this strategy.

## Abstract (tr)

İmmün sistemin kanser hücrelerini kontrol ve elimine etme özelliğine sahip olduğu gösterilmiştir. İmmün-kontrollü eliminasyonda kanser aşıları ve hematopoietik kök hücre naklini içeren sellüler terapiler bulunmaktadır. Adoptif T hücre transferi daha potent ve spesifiktir, hedef dışı toksisitesi azdır. Klinik çalışmalarda iki tür T hücresi test edilmektedir: T hücre reseptör ve kimerik antijen reseptör (KAR) modifiye T hücreleri. 1 Temmuz 2014’te Amerikan Gıda ve İlaç Dairesi anti-CD19 ŞAR modifiye T hücre tedavisini “çığır açan tedaviler” sınıfına almıştır. Bu yeni tedavi yöntemini ve etkilerini araştıran birçok çalışma yapılmıştır. Bu derleme adoptif immünoterapinin geçmişini, ŞAR modifiye T hücrelerini, üretim sürecini, klinik ve preklinik çalışmaları özetlemektedir.

## INTRODUCTION

Poor salvage chemotherapy success rates for refractory hematological diseases have necessitated novel approaches. Adoptive T-cell transfer has gained significant interest and clinical usage in hematology because of the off target effects of allogeneic stem cell transplantation and life threatening graft versus host disease (GVHD). Therefore, research efforts have sought to generate more specific T cells with higher toxicity to tumors and not healthy targets. To achieve curative potential, T cell immunotherapy combines potency, specificity and persistence [1]. Early approaches to adoptive T cell immunotherapy were based on the graft-versus-leukemia (GVL) effect mediated by donor lymphocyte infusion (DLI) hematopoietic stem cell transplantation (HSCT) and the therapeutic infusion of ex vivo expanded tumor-infiltrating lymphocytes (TILs) in combination with lymphodepletion for the treatment of advanced melanoma. However, DLI is usually associated with life-threatening forms of GVHD, and TILs require time-consuming procedures with unsuccessful results [2,3]. To overcome these drawbacks, genetically modified effector T cells have been developed as an alternative approach. In hematological malignancies, engineered T cell receptors (TCRs) and chimeric antigen receptors (CARs) are new powerful T-cell based immune therapies that target specific antigens. CAR T cells have been used successfully in the treatment of solid and hematological malignancies recently. In the following sections, the history of adoptive immunotherapy, TCR gene therapy, CART cell production, and preclinical and clinical studies will be discussed.

## THE ROLE OF T CELLS IN CANCER AND T CELLL RECEPTOR GENE THERAPY

In 1909, Paul Ehrlich first proposed that the immune defense system identifies and eliminates tumor cells [4]. However, recent studies revealed that the immune response may be ineffective against tumor development due to immunological tolerance and anergy [5]. Cancer immunoediting consists of three stages: elimination, equilibrium and escape. In the elimination stage, cancer is eliminated by intact innate and adaptive immunity, whereas in the equilibrium stage, variant tumor cells that develop genetic instability survive despite the immune attacks. Uncontrolled proliferation of variant tumor cells occurs in the escape stage [6].

In 1890, William B Coley observed that patients with malignancies respond to the intratumoral inoculation of live bacterial organisms or bacterial toxins that cause tumors to express unique proteins that could trigger an immune response [7]. Since the beginning of the 20th century, research has shown that most cancer cells carry overexpressed tumor-associated or tumor-specific antigens that are not present on healthy cells; this feature has led to the successful application of adoptive T-cell transfer. The discovery of T-cell growth factor, in vitro T-cell culture and the role of lymphodepletion have led to T-cell based therapy studies [8]. The first successful study on T-cell transfer immunotherapy using autologous TILs was performed in advanced melanoma in 1990 [9]. Since tumor infiltrating lymphocyte isolation was first attempted, in vitro expansion and re-infusion have been shown to be time-consuming and produce transient anti-tumor effects, and genetic engineering methods have been applied to create specific T cell-generated TCRs.

**Figure 1:**
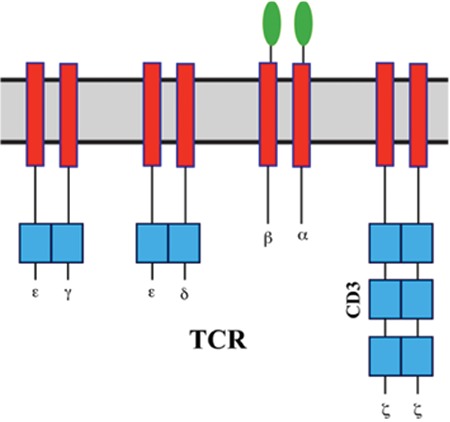
T cell receptor (adapted from Wieczorek and Uharek [10]).

The TCR is a heterodimer that carries information for defined tumor antigens and is formed by alpha and beta chains associated with a CD3 complex (Figure 1) [10]. TCR technology has advantages as a redirected T-cell therapy. Ideal effector T cells match with selected tumor target antigens through HLA recognition. The natural mechanism of T-cell immunity is associated with a low risk of cytokine release syndrome. The major difficulties that need to be overcome are the low surface expression of TCRs, HLA dependency, the and short persistence of transferred T-cells in vivo [11]. In thymic selection during the development of T cells, a few mutated proteins are encoded by cancer-causing genetic mutations (driver mutations), the large proportion of tumor antigens are self antigens, and T cells have low affinity for self antigens [12]. To create a higher avidity, selected TCRs from immunized human HLA transgenic mice with relevant epitopes are used along with insertion of targeted mutations in the complementary-determining region 2 or 3 (CDR2 or 3) in the variable regions of the TCR alpha/beta chains. These modified TCRs interact with the HLA/epitope complex [13]. However, TCRs can create unwanted alpha/beta heterodimers between the new and endogenous TCR alpha/beta chains in a process called mispairing, which results in low avidity [14]. TCR-modified T cells adapted for solid tumors have not been successful in most studies (Table 1) [10].

## CHIMERIC ANTIGEN RECEPTORS

**Figure 2:**
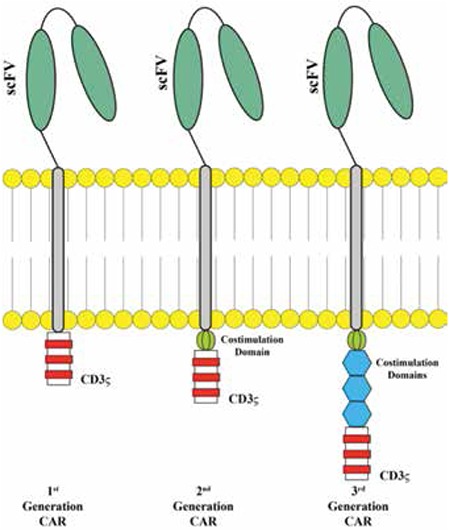
Generations of CART cells (adapted from Porter et al. [55]).

The genetic modification of T cells with CARs represents a breakthrough for gene engineering in hematological malignancies. The first CAR concept originated from the cloning of the TCR CD3 ζ-chain that was found to activate T cells independently [18]. First-generation CARs included only a single-chain variable fragment (scFv) that was constructed from the variable heavy and variable light sequences of a monoclonal antibody (mAb) specific for a tumor cell surface molecule and the cytoplasmic CD3 ζ-chain signaling domain. The initial studies were conducted in patients with HIV infection with prolonged survival [19]. In the first-generation cancer studies, CAR T cells did not proliferate in vivo and persistence was transient or the T cells were present at very low frequencies [20]. Based on a second genetic modification, CARs possess an antibody-based extracellular receptor structure that binds to target cells along with intracellular activating domains. Costimulatory protein receptors (e.g., CD28, CD137 (4-1BB), ICOS, CD134 (OX40), CD27, or CD244) were added to the cytoplasmic tail of the CAR in the second- and third-generation CARs [21] (Figure 2). Second-generation CARs are constructed with one costimulatory molecule while third-generation CARs contain more than one additional costimulatory molecule. The antitumor effect of CAR-T cells varies due to differences in the cytoplasmic domain and the extracellular domain’s ability to recognize a different epitope of the same antigen with different affinities for each CAR construct [22]. Whether the addition of secondary costimulation as in third-generation CARs obtains more efficacy is still an unanswered question [23]. CARs have several advantages: initiation of reliable high-potency signals, HLA independency, no requirement for antigen processing, and no competition for CD3. The number of target molecules on tumor cells that bind to CARs is greater than the number of major histocompatibility complex (MHC)/peptide complexes, and the scFv has a higher binding affinity for antigens than the TCRs [24]. Recently, Oren et al. compared the functional properties of engineered T cells expressing native low-affinity αβ-TCR chains with high-affinity TCR-like Ab-based CARs targeting the same specificity and suggested that the upper affinity threshold should be used to mediate effective functional outcomes of engineered T cells [25]. The major disadvantage of CARs is the massive cytokine release induced by binding and the immunogenicity of the mouse-derived scFv portion of the CAR complex, which may result in immune responses and the clearance of CAR T cells. In addition to that, intracellular molecules cannot be recognized [26].

## CHIMERIC ANTIGEN RECEPTOR T CELL MANUFACTURING

Gene transfer technology has rapidly developed; however, the clinical production of CARs for therapy is restricted to specialized, licensed manufacturing facilities with stringent rules (Good Manufacturing Process). In vitro culture systems for T cell expansion are used to manufacture large quantities of engineered T cells. The average production time to generate large numbers of unselected CD4 and CD8 T cells required for therapy is 10-14 days in clean rooms. First, peripheral blood mononuclear cells are isolated from the patient using leukapheresis, and T cells are selected by anti-CD3/anti-CD28 paramagnetic beads. Recent studies have demonstrated that less differentiated T cells have superior engraftment and antitumor activity [27]. In particular, CD8 T central memory cells can be modified with tumor-specific CARs [28]. T cells are then transduced with a CAR-encoding viral vector. Two vector systems, retroviral or lentiviral vectors, can be used to transfer CAR-coding genes into T cells. Retroviral vectors have permanent gene expression; however, the transduction can be performed only on efficiently dividing T cells. Lentiviral vectors can also integrate into nondividing cells. The disadvantages of viral vectors are the expense and experience required for production. Transposon systems such as Sleeping Beauty 100X (SB100X) or PiggyBac (PB) are new methods for genetic modification of T cells with high gene expression; they are simple and inexpensive and have large cargo capacity and low immunogenicity [10]. T cells are expanded in culture by stimulating them using the anti-CD3 clone OKT3 with cytokines like IL-2, IL-7, and IL-15. Moreover, in vivo persistence can be achieved by the overexpression of antiapoptotic proteins such as Bcl-2 or Bcl-xL. An adequate number of CAR T cells, which remains unknown, are then transferred to the patient using host preparative lymphodepletion regimens based on drugs and techniques to deplete Tregs, such as cyclophosphamide, fludarabine, low-dose irradiation, gemcitabine, denileukin diftitox, azacitidine, or decitabine [10,29,30]. CARs on T cells bind to their antigen on the tumor, and activation is controlled by the intracytoplasmic domains within the CAR. Tumor killing can be mediated by the direct cytotoxicity of the CD8+ CAR T cells with granzyme and perforin or cytokines released by CD4+ CAR T cells that bypass the MHC. Long-term eradication and prevention can be achieved by memory CAR T cells from a single infusion [31].

## STUDIES INVOLVING CHIMERIC ANTIGEN RECEPTOR T THERAPY

The ideal targets for CAR-modified T cells are expressed on tumor cells but are not expressed on normal cells. CD19 and CD20 are attractive targets due to their specificity for the B cell linage [32]. The first-generation CARs were not sufficient to produce a durable immune response; they rapidly underwent apoptosis after stimulation [33]. 19z CAR T cells were expanded on CD19+CD80+IL15+ cells and eradicated established systemic Raji tumors in 50% of SCID-beige mice [34]. Second-generation CARs that express CD28-containing costimulation in the CD19+CD80/CD86-ALL SCID-beige tumor model showed superior in vivo tumor activity and T cell function. CD22 is also under investigation and shows potential [35]. Imai et al. showed that in vivo anti-CD19 chimeric receptors containing the 4-1BB signal transduction domain had powerful antileukemic activity, destroying CD19+ acute lymphoblastic leukemia (ALL) cell lines in an in vivo microenvironment [33]. Target discovery for T cell leukemias and myeloid leukemias is problematic because blasts express the same antigens as normal hematopoietic stem cells [36]. For myeloid leukemias, CARs directed against CD123 have demonstrated efficacy in preclinical models; however, vascular endothelial cells also express CD123, which requires more investigation before clinical application [37]. Kenderian et al. stated that anti-CD33-specific CAR T cells exhibited significant effector functions in vitro and resulted in eradication of leukemia and prolonged survival in acute myeloid leukemia (AML) xenografts [38]. In multiple myeloma, CAR-engineered natural killer cells that targeted CS-1 protein displayed enhanced cytolysis in vitro [39].

The translation of this therapy to clinical settings involves various antigens and malignancies, and most trials have focused on B cell malignancies with B cell antigens CD19 and CD20 as the targets [40]. The first case report of CD19+ CAR T cells was published in 2011 by Porter et al. in relapsed refractory chronic lymphoid leukemia [41]. In that study, 3x108 T cells were transduced using a lentiviral vector, and the patient exhibited complete remission after 10 months. The largest dose-optimization trial involved 27 chronic lymphocytic leukemia (CLL) patients and found no difference between two doses of CAR T cells (<5x107 versus >5x107) with a complete response rate of 40% of patients [42]. In another study, CAR-modified T cells were shown to persist for more than 3 years with an initial response rate of 57% and complete remission of 29%, which was more favorable as compared to ibrutinib (an overall response rate of 71% but a complete remission rate of 2.4%) [43]. In B cell ALL (B-ALL), Davila et al. reported on 16 relapsed or refractory cases that were treated with 19-28z-expressing CAR T cells with an overall complete response rate of 88%, as compared to 44% with salvage chemotherapy. CAR T cells persisted for 2-3 months, and almost half of the patients proceeded to allogeneic stem cell transplantation [44]. In 30 ALL patients treated with CD19 CAR T cells, a 6-month event-free survival of 67% and overall survival of 78% were achieved, and ongoing remission for up to 2 years was possible without transplantation [45]. The underlying causes of the limited clinical efficacy of the CAR T cells in patients with CLL compared to B-ALL include the limited persistence of CAR T cells in CLL patients, the inhibitory effect of the tumor microenvironment in CLL, the lymph node-based nature of CLL, and the lower tumor burden at treatment in patients with B-ALL [40].

Patients with B cell malignancy were first treated with modified autologous CD20-specific T cells in 2008 by investigators from the Fred Hutchinson Cancer Research Center and the City of Hope National Medical Center. T cells persisted for up to 9 weeks with 7 patients with indolent or mantle cell lymphoma achieving partial response (1 patient), stable disease (4 patients), or complete response (2 patients) [46]. In 2014, an anti-CD19 chimeric antigen receptor trial for chemotherapy-refractory diffuse large B cell lymphoma and indolent B cell malignancies was published by Kochenderfer et al.; they demonstrated that 8 of 15 patients had complete response with 1-5x106 CAR T cells transduced by gammaretrovirus [47]. The targets for CAR therapy in multiple myeloma can be CD138, CD38, CD56, and CS1. Unlike CD19, these targets are coexpressed on other important cell types and result in unacceptable on-target, off-tumor toxicity. The first AML trial targeted the LeY antigen, and only 1 of 4 patients had 23 months of stable disease following therapy [48]. Contrary to preclinical studies, the CD33 antigen as a target was not proven to be safe due to the high level of toxicity against normal hematopoietic cells [49]. Phase I clinical trials involving CD123 targeting by mAbs and immunotoxins have produced only minor clinical responses, suggesting the need to develop more powerful AML strategies [50]. Table 2 shows the CAR T cell therapies in hematological malignancies [51].

## ADVERSE EFFECTS OF CHIMERIC ANTIGEN RECEPTOR T CELL THERAPY

As with all therapies, the toxicity from CAR T cells may be classified as on-target or off-target. The most common toxicity is cytokine release syndrome (CRS). In most cases, CRS is correlated with antitumor activity, and patients exhibit a range of symptoms from high fever, hypoxia, and hypotension to mild flu symptoms. The increased cytokines, particularly IL-6 and TNF-α, are produced by dying B cells, tumor cells, or macrophages [51]. Grupp et al. reported that the IL-6 receptor-blocking monoclonal antibody tocilizumab may ameliorate CRS in steroid-refractory circumstances without compromising T cell efficacy [59]. CRS was reported to occur in 6/13 patients with high complete response rates with tocilizumab as an alternative treatment option. The C-reactive protein level has been shown to be an indicator of severe CRS [45]. Another off-target adverse effect is tumor lysis syndrome, which is due to rapid and massive destruction of tumor cells. Macrophage activation syndrome is another life-threatening off-target effect of systemic inflammatory symptoms and pancytopenia, although the mechanisms are still unknown [42]. Several patients in CD19-CAR trials experienced reversible obtundation, seizures, aphasia, and mental status changes, possibly due to systemic cytokines crossing the blood-brain barrier [51]. B cell aplasia is an expected result of CD19-directed therapies and can be managed by γ-globulin replacement therapy. Persistent B cell aplasia results in an increased risk of infections [52].

## FUTURE DIRECTIONS

Adoptive T cell transfer has been used for the treatment of malignant diseases and may be regarded as an anticancer biopharmaceutical. A biopharmaceutical is defined as a product that is originally natural or derived from biological sources with industrial additions [62]. The main goals of T cell engineering are tumor antigen targeting and an increase in antitumor functions [1]. CAR T cell therapies are powerful breakthrough therapies, but several challenges need to be addressed. The optimal design of CARs remains an area of investigation. To be useful in other disease types, tumor-specific targets must be identified in solid tumors. T cell trafficking to the tumor microenvironment is critical in the moderate success against solid cancers [63]. To minimize severe toxicity, standardized approaches to the management of CRS should be applied [64]. B cell aplasia is still a problem with long-term exposure and may have an economic impact on health care. Once the B cell malignancy has been eradicated, anti-CD19-CAR T cells should be ablated to maintain normal B cell activity. A suicide system has been developed to eliminate gene-modified T cells when they display unwanted toxicities, such as the thymidine kinase gene of the herpes simplex virus [65]. Relapse remains a challenge and may be prevented with optimization of CAR design. Finally, in order for the therapy to become routinely used, automation and robotic culture technologies should be performed during the manufacturing process instead of manual cell culture technologies [66].

The induction of adoptive immunotherapy using CAR T cells has been successful in clinical trials, and the final goal is to induce durable immunity against disease progression without severe adverse effects. Whether this treatment option will replace HSCT or be used as a bridge to HSCT in the near future is still an unanswered question.

## Figures and Tables

**Table 1:**
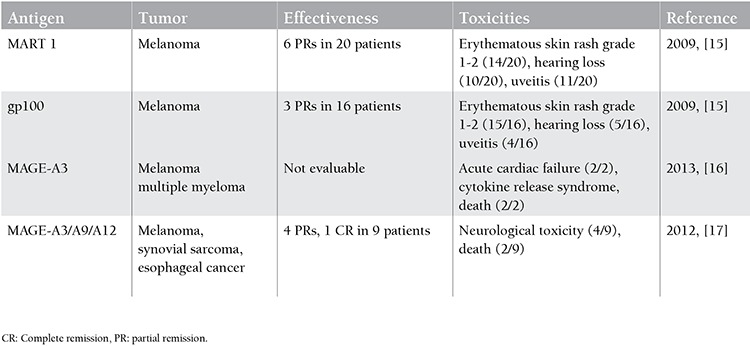
T cell receptor clinical studies.

**Table 2:**
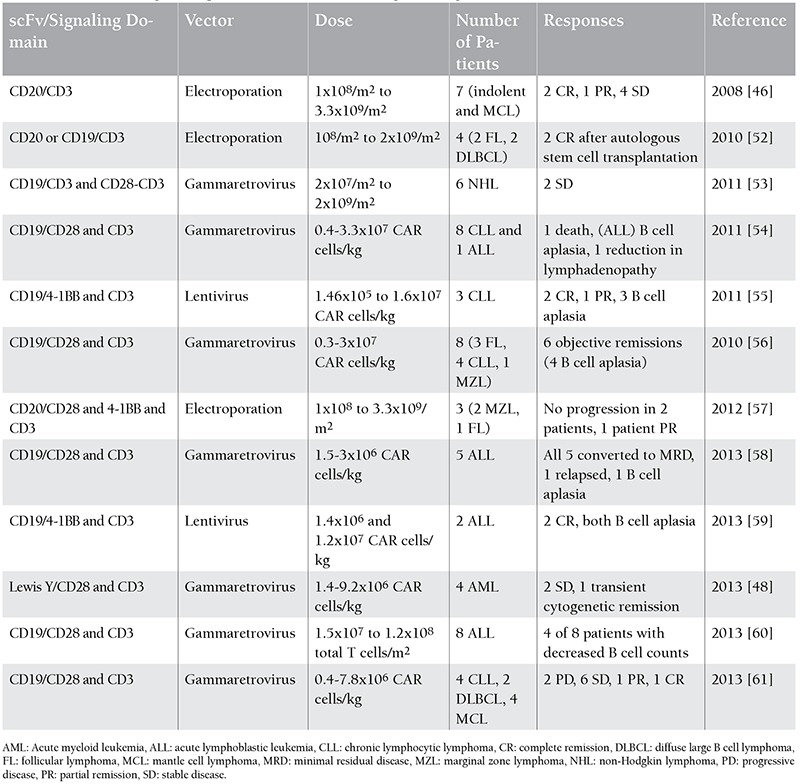
Chimeric antigen receptor T cell trials in hematological malignancies [55].

## References

[1] Sadelain M (2014). CAR T cell therapy: the CD19 paradigm.

[2] Miller JS, Warren EH, Ritz J, Shlomchik WD, Murphy WJ, Barrett AJ, Kolb HJ, Giralt S, Bishop MR, Blazar BR, Falkenburg JH (2010). NCI First International Workshop on the Biology, Prevention, and Treatment of Relapse After Allogeneic Hematopoietic Stem Cell Transplantation: Report from the Committee on the Biology Underlying Recurrence of Malignant Disease Following Allogeneic HSCT: Graft-versus-Tumor/Leukemia Reaction. Biol. Blood Marrow Transplant.

[3] Rosenberg SA, Dudley ME (2009). Adoptive cell therapy for the treatment of patients with metastatic melanoma. Curr Opin Immunol.

[4] Ehrlich P (1909). Über den jetzigen Stand der Karzinomforschung. Ned Tijdschr Geneeskd.

[5] Kim R, Emi M, Tanabe K (2007). Cancer immunoediting from immune surveillance to immune escape. J Immunol.

[6] Dunn GP, Old LJ, Schreiber RD (2004). The three Es of cancer immunoediting. Ann Rev Immunol.

[7] Chang AE, Shu S (1996). Current status of adoptive immunotherapy of cancer. Crit Rev Oncol Hematol.

[8] Shankaran V, Ikeda H, Bruce AT, White JM, Swanson PE, Old LJ, Schreiber RD (2001). IFNγ and lymphocytes prevent primary tumour development and shape tumour immunogenicity. Nature.

[9] Rosenberg SA, Aebersold P, Cornetta K, Kasid A, Morgan RA, Moen R, Karson EM, Lotze MT, Yang JC, Topalian SL, Merino MJ, Culver K, Miller AD, Blaese RM, Anderson WF (1990). Gene transfer into humans — immunotherapy of patients with advanced melanoma, using tumor-infiltrating lymphocytes modified by retroviral gene transduction. N Eng J Med.

[10] Wieczorek A, Uharek L (2013). Genetically modified T cells for the treatment of malignant disease. Transfus Med Hemother.

[11] Jensen MC, Riddell SR (2014). Design and implementation of adoptive therapy with chimeric antigen receptor-modified T cells. Immunol Rev.

[12] June CH (2007). Principles of adoptive T cell cancer therapy J Clin Inv.

[13] Stauss HJ (1999). Immunotherapy with CTLs restricted by nonself MHC. Immunol Today.

[14] Cohen CJ, Li YF, El-Gamil M, Robbins PF, Rosenberg SA, Morgan RA (2007). Enhanced antitumor activity of T cells engineered to express T-cell receptors with a second disulfide bond. Cancer Res.

[15] Johnson LA, Morgan RA, Dudley ME, Cassard L, Yang JC, Hughes MS, Kammula US, Royal RE, Sherry RM, Wunderlich JR, Lee CC, Restifo NP, Schwarz SL, Cogdill AP, Bishop RJ, Kim H, Brewer CC, Rudy SF, VanWaes C, Davis JL, Mathur A, Nathan DA, Laurencot CM, Rosenberg SA (2009). Gene therapy with human and mouse T-cell receptors mediates cancer regression and targets normal tissues expressing cognate antigen. Blood.

[16] Linette GP, Stadtmauer EA, Maus MV, Rapoport AP, Levine BL, Emery L, Litzky L, Bagg A, Carreno BM, Cimino PJ, Binder-Scholl GK, Smethurst DP, Gerry AB, Pumphrey NJ, Bennett AD, Brewer JE, Dukes J, Harper J, Tayton-Martin HK, Jakobsen BK, Hassan NJ, Kalos M, June CH (2013). Cardiovascular toxicity and titin cross-reactivity of affinity- enhanced T cells in myeloma and melanoma. Blood.

[17] Morgan RA, Chinnasamy N, Abate-Daga D, Gros A, Robbins PF, Zheng Z, Dudley ME, Feldman SA, Yang JC, Sherry RM, Phan GQ, Hughes MS, Kammula US, Miller AD, Hessman CJ, Stewart AA, Restifo NP, Quezado MM, Alimchandani M, Rosenberg AZ, Nath A, Wang T, Bielekova B, Wuest SC, Akula N, McMahon FJ, Wilde S, Mosetter B, Schendel DJ, Laurencot CM, Rosenberg SA (2013). Cancer regression and neurological toxicity following anti-MAGE-A3 TCR gene therapy. J Immunother.

[18] Irving BA, Weiss A (1991). The cytoplasmic domain of the T cell receptor zeta chain is sufficient to couple to receptor-associated signal transduction pathways. Cell.

[19] Mitsuyasu RT, Anton PA, Deeks SG, Scadden DT, Connick E, Downs MT, Bakker A, Roberts MR, June CH, Jalali S, Lin AA, Pennathur-Das R, Hege KM (2000). Prolonged survival and tissue trafficking following adoptive transfer of CD4 z gene-modified autologous CD4+ and CD8+ T cells in HIV-infected subjects. Blood.

[20] Jensen MC, Clarke P, Tan G, Wright C, Chung-Chang W, Clark TN, Zhang F, Slovak ML, Wu AM, Forman SJ, Raubitschek A (2000). Human T lymphocyte genetic modification with naked DNA. Mol Ther.

[21] Kalos M, Levine BL, Porter DL, Katz S, Grupp SA, Bagg A, June CH (2011). T cells with chimeric antigen receptors have potent antitumor effects and can establish memory in patients with advanced leukemia. Sci Transl Med.

[22] Chmielewski M, Hombach A, Heuser C, Adams GP, Abken H (2004). T cell activation by antibody-like immunoreceptors: increase in affinity of the single-chain fragment domain above threshold does not increase T cell activation against antigen-positive target cells but decreases selectivity. J Immunol.

[23] Zhong XS, Matsushita M, Plotkin J, Riviere I, Sadelain M (2010). Chimeric antigen receptors combining 4-1BB and CD28 signaling domains augment PI3kinase/AKT/Bcl-XL activation and CD8+ T cell-mediated tumor eradication. Mol Ther.

[24] Du X, Beers R, FitzGerald DJ, Pastan I (2008). Differential cellular internalization of anti-CD19 and -CD22 immunotoxins results in different cytotoxic activity. Cancer Res.

[25] Oren R, Hod-Marco M, Haus-Cohen M, Thomas S, Blat D, Duvshani N, Denkberg G, Elbaz Y, Benchetrit F, Eshhar Z, Stauss H, Reiter Y (2014). Functional comparison of engineered T cells carrying a native TCR versus TCR-like antibody-based chimeric antigen receptors indicates affinity/avidity thresholds. J Immunol.

[26] Ramos CA, Dotti G (2011). Chimeric antigen receptor (CAR)-engineered lymphocytes for cancer therapy. Expert Opin Biol Ther.

[27] Gattinoni L, Klebanoff CA, Restifo NP (2012). Paths to stemness: building the ultimate antitumour T cell. Nat Rev Cancer.

[28] Stemberger C, Dreher S, Tschulik C, Piossek C, Bet J, Yamamoto TN, Schiemann M, Neuenhahn M, Martin K, Schlapschy M, Skerra A, Schmidt T, Edinger M, Riddell SR, Germeroth L, Busch DH (2012). Novel serial positive enrichment technology enables clinical multiparameter cell sorting. PLoS One.

[29] Lamers CH, Elzakker P, Langeveld SC, Sleijfer S, Gratama JW (2006). Process validation and clinical evaluation of a protocol to generate gene-modified T lymphocytes for imunogene therapy for metastatic renal cell carcinoma: GMP-controlled transduction and expansion of. patient’s T lymphocytes using a carboxy anhydrase IX-specific scFv transgene. Cytotherapy.

[30] Lamers CH, Elzakker P, Steenbergen SC, Luider BA, Groot C, Krimpen BA, Vulto A, Sleijfer S, Debets R, Gratama JW (2013). Long-term stability of T-cell activation and transduction components critical to the processing of clinical batches of gene-engineered T cells. Cytotherapy.

[31] Sadelain M, Riviere I, Brentjens R (2003). Targeting tumours with genetically enhanced T lymphocytes. Nat Rev Cancer.

[32] Scheurmann RH, Racila E (1995). CD19 antigen in leukemia and lymphoma diagnosis and immunotherapy. Leuk Lymphoma.

[33] Imai C, Mihara K, Andreansky M, Nicholson IC, Pui CH, Geiger TL, Campana D (2004). Chimeric receptors with 4-1BB signaling capacity provoke potent cytotoxicity against acute lymphoblastic leukemia. Leukemia.

[34] Brentjens RJ, Latouche JB, Santos E, Marti F, Gong MC, Lyddane C, King PD, Larson S, Weiss M, Rivière I, Sadelain M (2003). Eradication of systemic B-cell tumors by genetically targeted human T lymphocytes co-stimulated by CD80 and interleukin-15. Nat Med.

[35] Haso W, Lee DW, Shah NN, Stetler-Stevenson M, Yuan CM, Pastan IH, Dimitrov DS, Morgan RA, FitzGerald DJ, Barrett DM, Wayne AS, Mackall CL, Orentas RJ (2013). Anti-CD22 chimeric antigen receptors targeting B-cell precursor acute lymphoblastic leukemia. Blood.

[36] Gasiorowski RE, Clark GJ, Bradstock K, Hart DN (2014). Antibody therapy for acute myeloid leukemia. Br J Haematol.

[37] Pizzitola I (2014). Chimeric antigen receptors against CD33/CD123 antigens efficiently target primary acute myeloid leukemia cells in vivo. Leukemia.

[38] Kenderian SS, Ruella M, Shestova O, Klichinsky M, Aikawa V, Morrissette JJD, Scholler J, Song D, Porter DL, Carroll M, June CH, Gill S ((in press)). CD33 specific chimeric antigen receptor T cells exhibit potent preclinical activity against human acute myeloid leukemia.

[39] Chu J, Deng Y, Benson DM, He S, Hughes T, Zhang J, Peng Y, Mao H, Yi L, Ghoshal K, He X, Devine SM, Zhang X, Caligiuri MA, Hofmeister CC, Yu J (2014). CS1-specific chimeric antigen receptor CAR-engineered natural killer cells enhance in vitro and in vivo antitumor activity against human multiple myeloma. Leukemia.

[40] Davila ML, Bouhassira DC, Park JH, Curran KJ, Smith EL, Pegram HJ, Brentjens R (2014). Chimeric antigen receptors for the adoptive T cell therapy of hematologic malignancies. Int J Hematol.

[41] Porter DL, Levine BL, Kalos M, Bagg A, June CH (2011). Chimeric antigen receptor-modified T cells in chronic lymphoid leukemia. N Engl J Med.

[42] Porter DL, Kalos M, Frey NV, Grupp SA, Loren AW, Jemision C, Gilmore J, McConville H, Capobianchi J, Lledo L, Chew A, Shen A, Wood PA, Litchman M, Zheng Z, Levine BL, June CH (2013). Randomized, phase II dose optimization study of chimeric antigen receptor modified T cells directed against CD19 (CTL019) in patients with relapsed, refractory CLL. Abstract 873. ASH Annual Meeting.

[43] Brown JR, Porter DL, O’Brien SM (2014). Novel treatments for chronic lymphocytic leukemia and moving forward. Am Soc Clin Oncol Educ Book.

[44] Davila ML, Riviere I, Wang X, Bartido S, Park J, Curran K, Chung SS, Stefanski J, Borquez-Ojeda O, Olszewska M, Qu J, Wasielewska T, He Q, Fink M, Shinglot H, Youssif M, Satter M, Wang Y, Hosey J, Quintanilla H, Halton E, Bernal Y, Bouhassira DC, Arcila ME, Gonen M, Roboz GJ, Maslak P, Douer D, Frattini MG, Giralt S, Sadelain M, Brentjens R (2014). Efficacy and toxicity management of 19-28z CAR T cell therapy in B cell acute lymphoblastic leukemia. Sci Transl Med.

[45] Maude SL, Frey N, Shaw PA, Aplenc R, Barrett DM, Bunin NJ, Chew A, Gonzalez VE, Zheng Z, Lacey SF, Mahnke YD, Melenhorst JJ, Rheingold SR, Shen A, Teachey DT, Levine BL, June CH, Porter DL, Grupp SA (2014). Chimeric antigen receptor T cells for sustained remissions in leukemia. N Eng J Med.

[46] Till BG, Jensen MC, Wang J, Chen EY, Wood BL, Greisman HA, Qian X, James SE, Raubitschek A, Forman SJ, Gopal AK, Pagel JM, Lindgren CG, Greenberg PD, Riddell SR, Press OW (2008). Adoptive immunotherapy for indolent non-Hodgkin lymphoma and mantle cell lymphoma using genetically modified autologous CD20-specific T cells. Blood.

[47] Kochenderfer JN, Dudley ME, Kassim SH, Somerville RPT, Carpenter RO, Stetler-Stevenson M, Yang JC, Phan GQ, Hughes MS, Sherry RM, Raffeld M, Feldman S, Lu L, Li YF, Ngo LT, Goy A, Feldman T, Spaner DE, Wang ML, Chen CC, Kranick SM, Nath A, Nathan DN, Morton KE, Toomey MA, Rosenberg SA ((in press)). Chemotherapy-refractory diffuse large B-cell lymphoma and indolent B-Cell malignancies can be effectively treated with autologous T cells expressing an anti-CD19 chimeric antigen receptor.

[48] Ritchie DS, Neeson PJ, Khot A, Peinert S, Tai T, Tainton K, Chen K, Shin M, Wall DM, Hönemann D, Gambell P, Westerman DA, Haurat J, Westwood JA, Scott AM, Kravets L, Dickinson M, Trapani JA, Smyth MJ, Darcy PK, Kershaw MH, Prince HM (2013). Persistence and efficacy of second generation CAR T cell against the LeY antigen in acute myeloid leukemia. Mol Ther.

[49] Larson RA, Sievers EL, Stadtmauer EA, Löwenberg B, Estey EH, Dombret H, Theobald M, Voliotis D, Bennett JM, Richie M, Leopold LH, Berger MS, Sherman ML, Loken MR, Dongen JJ, Bernstein ID, Appelbaum FR (2005). Final report of the efficacy and safety of gemtuzumab ozogamicin (Mylotarg) in patients with CD33-positive acute myeloid leukemia in first recurrence. Cancer.

[50] Tettamanti S, Biondi A, Biagi E, Bonnet D (2014). CD123 AML targeting by chimeric antigen receptors: a novel magic bullet for AML therapeutics?. Oncoimmunology.

[51] Maus MV, Grupp SA, Porter D, June CH (2014). Antibody-modified T cells: CARs take the front seat for hematologic malignancies. Blood.

[52] Jensen MC, Popplewell L, Cooper LJ, DiGiusto D, Kalos M, Ostberg JR, Forman SJ (2010). Antitransgene rejection responses contribute to attenuated persistence of adoptively transferred CD20/CD19-specific chimeric antigen receptor redirected T cells in humans. Biol Blood Marrow Transplant.

[53] Savoldo B, Ramos CA, Liu E, Mims MP, Keating MJ, Carrum G, Kamble RT, Bollard CM, Gee AP, Mei Z, Liu H, Grilley B, Rooney CM, Heslop HE, Brenner MK, Dotti G (2011). CD28 costimulation improves expansion and persistence of chimeric antigen receptor-modified T cells in lymphoma patients. J Clin Invest.

[54] Brentjens RJ, Rivière I, Park JH, Davila ML, Wang X, Stefanski J, Taylor C, Yeh R, Bartido S, Borquez-Ojeda O, Olszewska M, Bernal Y, Pegram H, Przybylowski M, Hollyman D, Usachenko Y, Pirraglia D, Hosey J, Santos E, Halton E, Maslak P, Scheinberg D, Jurcic J, Heaney M, Heller G, Frattini M, Sadelain M (2011). Safety and persistence of adoptively transferred autologous CD19-targeted T cells in patients with relapsed or chemotherapy refractory B-cell leukemias. Blood.

[55] Porter DL, Levine BL, Kalos M, Bagg A, June CH (2011). Chimeric antigen receptor-modified T cells in chronic lymphoid leukemia. N Engl J Med.

[56] Kochenderfer JN, Wilson WH, Janik JE, Dudley ME, Stetler-Stevenson M, Feldman SA, Maric I, Raffeld M, Nathan DA, Lanier BJ, Morgan RA, Rosenberg SA (2010). Eradication of B-lineage cells and regression of lymphoma in a patient treated with autologous T cells genetically engineered to recognize CD19. Blood.

[57] Till BG, Jensen MC, Wang J, Qian X, Gopal AK, Maloney DG, Lindgren CG, Lin Y, Pagel JM, Budde LE, Raubitschek A, Forman SJ, Greenberg PD, Riddell SR, Press OW (2012). CD20-specific adoptive immunotherapy for lymphoma using a chimeric antigen receptor with both CD28 and 4-1BB domains: pilot clinical trial results. Blood.

[58] Brentjens R, Davila ML, Riviere I, Park J, Wang X, Cowell LG, Bartido S, Stefanski J, Taylor C, Olszewska M, Borquez-Ojeda O, Qu J, Wasielewska T, He Q, Bernal Y, Rijo IV, Hedvat C, Kobos R, Curran K, Steinherz P, Jurcic J, Rosenblat T, Maslak P, Frattini M, Sadelain M (2013). CD19-targeted T cells rapidly induce molecular remissions in adults with chemotherapy-refractory acute lymphoblastic leukemia. Sci Transl Med.

[59] Grupp SA, Kalos M, Barrett D, Aplenc R, Porter DL, Rheingold SR, Teachey DT, Chew A, Hauck B, Wright JF, Milone MC, Levine BL, June CH (2013). Chimeric antigen receptor-modified T cells for acute lymphoid leukemia. N Engl J Med.

[60] Cruz CR, Micklethwaite KP, Savoldo B, Ramos CA, Lam S, Ku S, Diouf O, Liu E, Barrett AJ, Ito S, Shpall EJ, Krance RA, Kamble RT, Carrum G, Hosing CM, Gee AP, Mei Z, Grilley BJ, Heslop HE, Rooney CM, Brenner MK, Bollard CM, Dotti G (2013). Infusion of donor-derived CD19-redirected virus-specific T cells for B-cell malignancies relapsed after allogeneic stem cell transplant: a phase 1 study. Blood.

[61] Kochenderfer JN, Dudley ME, Carpenter RO, Kassim SH, Rose JJ, Telford WG, Hakim FT, Halverson DC, Fowler DH, Hardy NM, Mato AR, Hickstein DD, Gea-Banacloche JC, Pavletic SZ, Sportes C, Maric I, Feldman SA, Hansen BG, Wilder JS, Blacklock-Schuver B, Jena B, Bishop MR, Gress RE, Rosenberg SA (2013). Donor-derived CD19-targeted T cells cause regression of malignancy persisting after allogeneic hematopoietic stem cell transplantation. Blood.

[62] Rader RA (2008). (Re)defining biopharmaceutical. Nat Biotechnol.

[63] Slaney CY, Kershaw MH, Darcy PK (2014). Trafficking of T cells into tumors. Cancer Res.

[64] Lee DW, Gardner R, Porter DL, Louis CU, Ahmed N, Jensen M, Grupp SA, Mackall CL (2014). Current concepts in the diagnosis and management of cytokine release syndrome. Blood.

[65] Ciceri F, Bonini C, Stanghellini MT, Bondanza A, Traversari C, Salomoni M, Turchetto L, Colombi S, Bernardi M, Peccatori J, Pescarollo A, Servida P, Magnani Z, Perna SK, Valtolina V, Crippa F, Callegaro L, Spoldi E, Crocchiolo R, Fleischhauer K, Ponzoni M, Vago L, Rossini S, Santoro A, Todisco E, Apperley J, Olavarria E, Slavin S, Weissinger EM, Ganser A, Stadler M, Yannaki E, Fassas A, Anagnostopoulos A, Bregni M, Stampino CG, Bruzzi P, Bordignon C (2009). Infusion of suicide-gene-engineered donor lymphocytes after family haploidentical haemopoietic stem-cell transplantation for leukaemia (the TK007 trial): a non-randomised phase I-II study. Lancet Oncol.

[66] Levine BL, June CH (2013). Perspective: assembly line immunotherapy. Nature.

